# Both gravistimulation onset and removal trigger an increase of cytoplasmic free calcium in statocytes of roots grown in microgravity

**DOI:** 10.1038/s41598-018-29788-7

**Published:** 2018-07-30

**Authors:** François Bizet, Veronica Pereda-Loth, Hugo Chauvet, Joëlle Gérard, Brigitte Eche, Christine Girousse, Monique Courtade, Gérald Perbal, Valérie Legué

**Affiliations:** 1 Université Clermont Auvergne, INRA, PIAF, F-63000 Clermont-Ferrand, France; 2 GSBMS, AMIS5288, University of Toulouse III, Toulouse, France; 3 UMR IAM, INRA, Université de Lorraine, 54280 Champenoux, France; 4 UMR EEF, INRA, Université de Lorraine, 54280 Champenoux, France; 5 Université Clermont Auvergne, INRA, GDEC, F- 63000 Clermont-Ferrand, France; 6 Université Pierre et Marie Curie, 75005 Paris, France

## Abstract

Gravity is a permanent environmental signal guiding plant growth and development. Gravity sensing in plants starts with the displacement of starch-filled plastids called statoliths, ultimately leading to auxin redistribution and organ curvature. While the involvement in gravity sensing of several actors such as calcium is known, the effect of statolith displacement on calcium changes remains enigmatic. Microgravity is a unique environmental condition offering the opportunity to decipher this link. In this study, roots of *Brassica napus* were grown aboard the International Space Station (ISS) either in microgravity or in a centrifuge simulating Earth gravity. The impact of short simulated gravity onset and removal was measured on statolith positioning and intracellular free calcium was assessed using pyroantimonate precipitates as cytosolic calcium markers. Our findings show that a ten-minute onset or removal of gravity induces very low statolith displacement, but which is, nevertheless, associated with an increase of the number of pyroantimonate precipitates. These results highlight that a change in the cytosolic calcium distribution is triggered in absence of a significant statolith displacement.

## Introduction

Gravity sensing in plants occurs in specialized cells called statocytes which are located in the shoot endodermis and in the central root cap^1^. Statocytes contain starch-filled plastids called statoliths. Being denser than the cytoplasm, the statolith position depends on the direction of gravity. Thus, it is assumed that statocytes constitute one of the plant gravity sensors in the starch-statolith hypothesis^2,3^. In roots, statocytes show an intriguing polarization of organelles. The nucleus is located at the shootward end of the cell whereas the endoplasmic reticulum (ER) is mostly at the rootward end^3^. The ER has long been suspected to be involved in the early gravity transduction pathway. In the 70 s, longitudinal sections of root apices highlighted compression of the rootward ER complex by sedimented statoliths^4–6^. This observation that the weight of statoliths is sufficient to locally deform the ER membranes was later confirmed using high-resolution electron tomography^7^. Accordingly, the ability of plants to orient their growth along the gravity vector, called gravitropism, was hypothesized to be the consequence of the pressure exerted by statoliths on the rootward ER complex. This widespread hypothesis was used to explain differences in root sensitivity to gravity observed at various inclination changes^8^. However recent investigations have highlighted that the gravitropism answer is independent of gravity intensity and thus that the mechanism behind gravity sensing in plants does not rely on a pressure applied by statoliths as a force sensor would do^9^. In this framework, the hypothesis of an inclination sensor able to detect statolith position rather than pressure was recently proposed^10^.

To date, several space experiments dealing with gravity sensing to date have focused on identifying a minimum dose (*i.e*. the product of gravity intensity with time) associated with gravitropic response^11–14^. These space experiments, coupled with Earth experiments using a clinostat, suggest that the gravitropic response is dependent upon the dose of gravity applied. These observations are fully compatible with a sensing based on statolith repositioning when thinking in terms of statolith avalanches^10^. The microgravity environment indeed offers the opportunity to nullify any pressure applied by statoliths and further tests the importance of statolith pressure in triggering the gravity transduction pathway. In the case of an inclination sensor detecting statolith positioning, a mechanism of direct or close interaction between statoliths and another cell component in triggering the gravity transduction pathway is expected. Several proteins located on plastids membranes are potential candidates for statolith position detection^15,16^. Moreover, statoliths are known to be displaced toward the shootward part of the statocytes in microgravity (*i.e*. toward the nucleus), presumably by an actin cytoskeleton-mediated process^17^. Thus, microgravity can also be used for removing contacts between sedimented statoliths and any cell component without any change in orientation.

Calcium is considered to be an important actor in response to environmental changes. However, the results seem to be contradictory concerning the role of Ca in the gravity signal transduction. Initially, the concentration of cytosolic free calcium, [Ca^2+^]_c,_ changes were initially not detected in *Arabidopsis* root caps^18^. More recent advances in Ca^2+^ imaging methods have enabled the detection of [Ca^2+^]_c_ in response to a gravistimulation^19^, and [Ca^2+^]_c_ increases were later confirmed in *Arabidopsis* roots^20^. The gravistimulation triggered by a modification of plant inclination induces two peaks of [Ca^2+^]_c_ changes during the minutes following gravistimulation in young *Arabidopsis* seedlings^21^. Using parabolic flights, Toyota and co-workers^22^ showed that the first transient [Ca^2+^]_c_ peak was caused by the change in orientation while the second sustained [Ca^2+^]_c_ peak was due to gravistimulation. Interestingly, these works suggest that the Ca^2+^ also triggers downstream changes, reflects auxin-triggered signaling during the gravitropism response which changes the orientation of seedling. Thus, space experiments offer the advantage of inducing gravity stimulus without any change in orientation of the seedling.

In this study, *Brassica napus* L. seedlings were grown aboard the International Space Station (ISS) either in simulated gravity (on a centrifuge with and acceleration equivalent to 1 *g*) or in microgravity. Modifications of statolith positioning were achieved by (i) removing gravity in roots grown on a centrifuge by stopping the simulated gravity, (ii) subjecting roots grown in microgravity to a centrifuge acceleration. The experiment took advantage of the fixative injection being automatically accomplished in the centrifuge without human intervention, therefore avoiding artefactual statolith repositioning. A 10 minute duration time was chosen so that the end point measurements were made after the triggering of the gravity transduction pathway. Our previous experiments, dedicated to finding the presentation time (*i.e*. the minimum duration of gravistimulus needed to induce a gravitropic response) for gravistimulated rapeseed seedlings, indicated that a time period of one to two minutes of gravistimulation is sufficient for triggering a gravitropic response^23^. In the same way, the experiments performed in parabolic flights highlighted transient intracellular calcium fluctuations within the first minute following gravistimuli^22^.

To access the effect of the position or displacement of statoliths on the trigger of calcium-dependant pathways, the changes in the amount of cytoplasmic free calcium were monitored by using a method specific for calcium precipitation in plants namely the pyroantimonate (PA) precipitation method^23^.

## Results

### Root growth and root apex orientation

**Figure 1:**
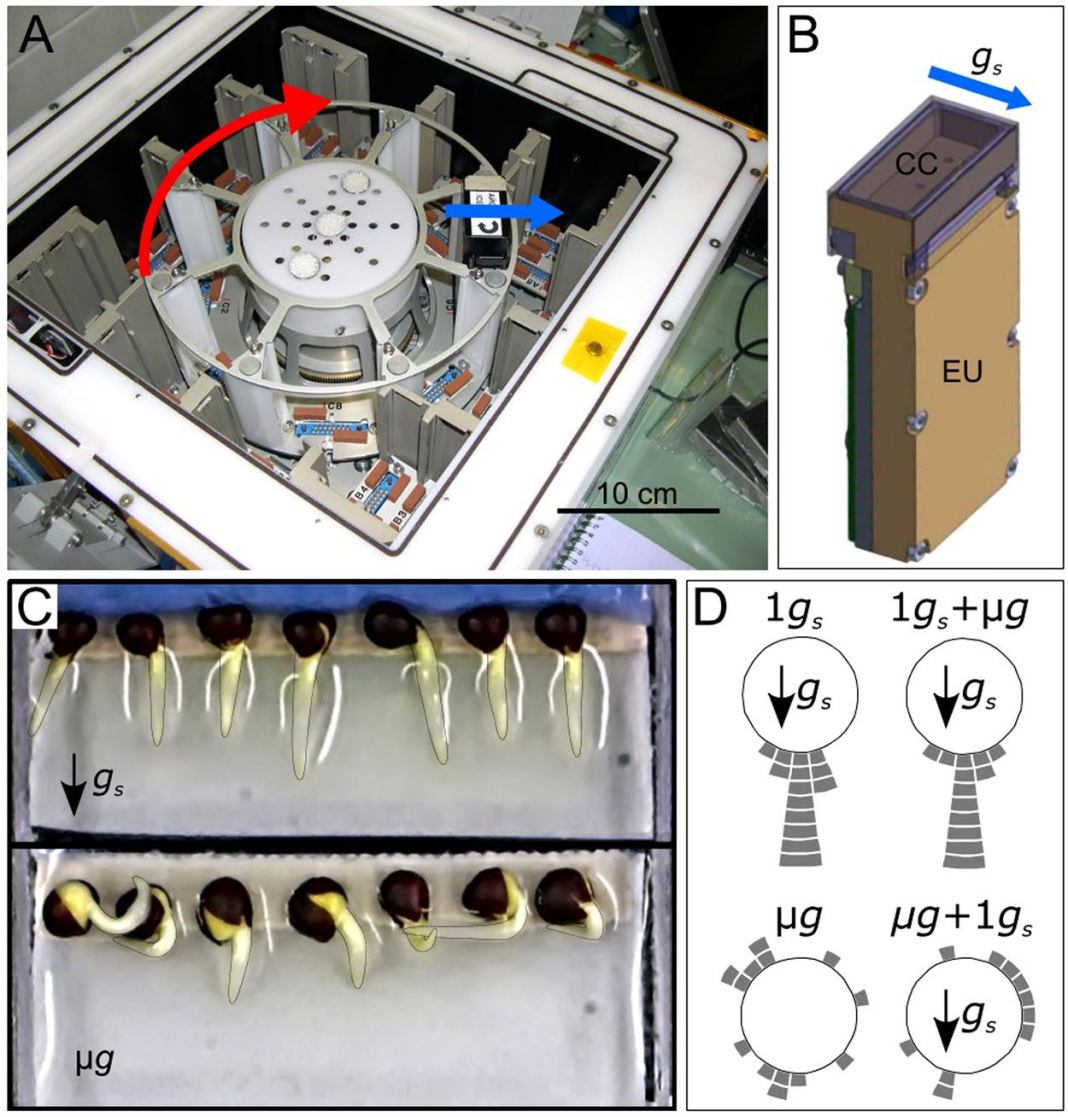
Kubik instrument, culture chambers and root apex orientation at the end of the experiment. (**A**) Inside of the Kubik facility highlighting the position of the experimental units (EUs) along the centrifuge rotor. (**B**) EU with a view on the culture chamber (CC) located at the top right-hand side. Water and fixatives pouches are hidden inside the EU. (**C**) Representative CCs after 40 h after hydration in simulated gravity (top) and microgravity (bottom) conditions. (**D**) Distribution of root apex orientation per class of 15° angle for each gravity condition (each tile stands for one root). The red arrow indicates the direction of rotation of the Kubik centrifuge. The blue arrows indicate the direction of the simulated gravity vector. See Table 1 for description of growth conditions.

*Brassica napus* seeds were germinated in culture chambers (CCs) set up in the Kubik facility (Fig. 1A,B). Forty hours after hydration in simulated gravity condition (1 *g*_*s*_), the mean length of *Brassica napus* primary root was 5.9 ± 0.2 mm (mean ± se, n = 44) (Table 1). No significant difference (P = 0.085) was found in primary root lengths between microgravity (µ*g*) and 1 *g*_*s*_. Root lengths were also statistically similar between the 10 minute of simulated gravity onset and removal (µ*g* + 1 *g*_*s*_ and 1 *g*_*s*_ + µ*g*) and their respective control (1 *g*_*s*_ and µ*g*).

**Table 1:** Root length of *Brassica napus* seedlings at the end of the experiment.

Condition	Root length (mm)	n
1 *g*_*s*_	5.7^a^ ± 0.3	11
1 *g*_*s*_ + µ*g*	6.7^a^ ± 0.5	11
µ*g*	5.4^a^ ± 0.3	13
µ*g* + 1 *g*_*s*_	5.5^a^ ± 0.4	9

*B. napus* seedlings were grown for 40 h in simulated gravity (1 *g*_s_); 39 h 50 min in simulated gravity followed by 10 minutes in microgravity (1*g*_s_ + µ*g*); 40 h in microgravity (µg); 39 h 50 min in microgravity followed by 10 minutes in simulated gravity (µ*g* + 1 *g*_s_). Data presented are mean ± se. By column, means followed by a same letter are not significantly different at a 5% level using a Student-Newmans Keuls test. Roots that were partially hidden by foreground elements in the pictures were not measured.

Figure 1D illustrates the angle distribution relative to the simulated gravity vector of all root apices at the end of the experiment. Roots growing in 1 *g*_*s*_ conditions were all directed toward the simulated gravity vector, while apices of roots growing in microgravity conditions spread over all directions (Fig. 1D). The observed distribution of root apex orientation in µ*g* + 1 *g*_*s*_ and in 1 *g*_*s*_ + µ*g*, were close to their respective control and with similar standard deviation (Supplementary Fig. S1). Thus, a change in the root apex orientation was not detected after the 10 minutes of simulated gravity onset and removal, suggesting that only early signalling events occurred within this period.

### Potassium pyroantimonate precipitates in root statocytes

**Figure 2:**
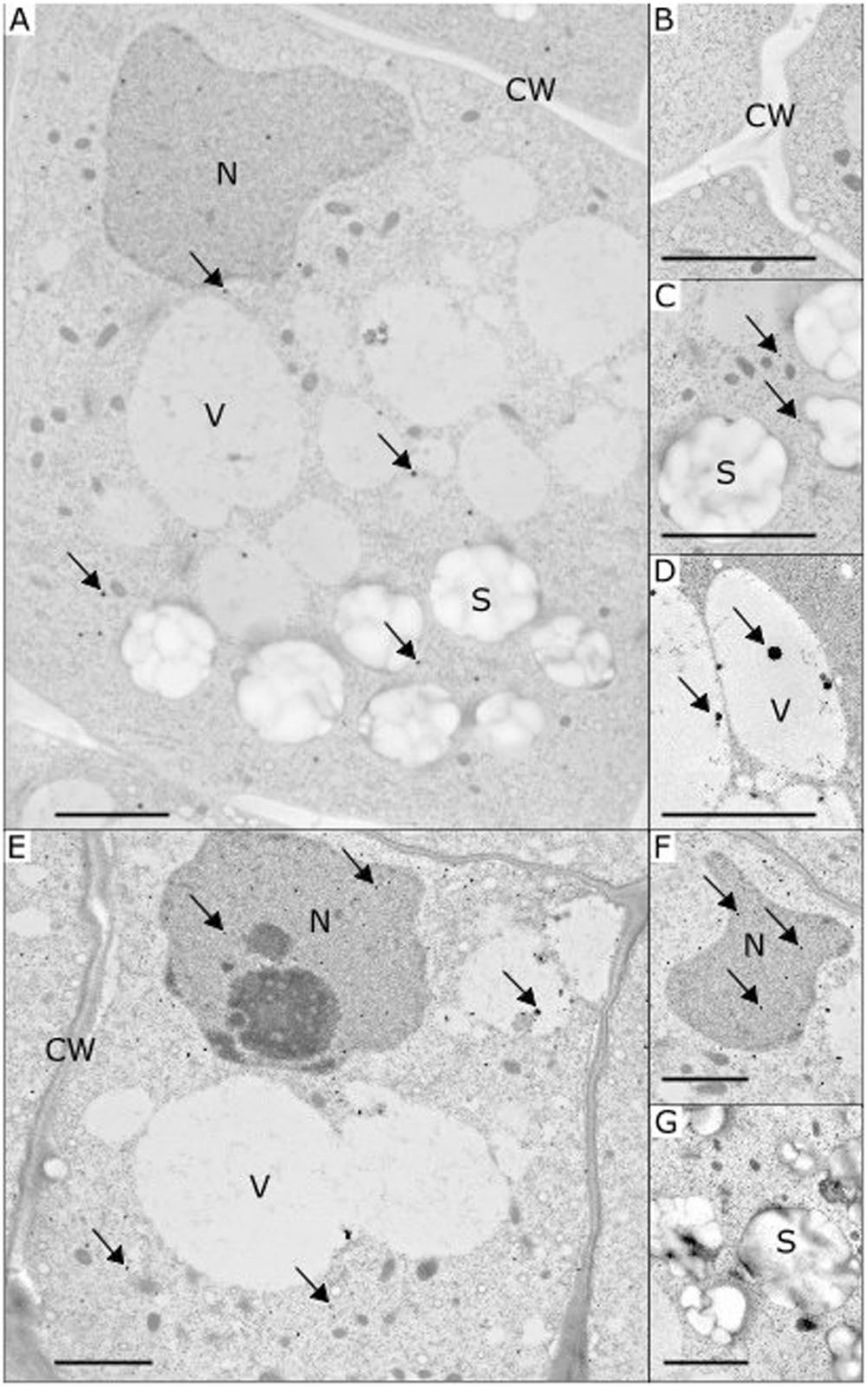
Representative transmission electron microscopy images of the root statocytes of *Brassica napus* seedlings with the presence of potassium pyroantimonate (PA) precipitates. Images are shown for seedlings grown in simulated gravity (**A**–**C**), microgravity (**D**) or after simulated gravity removal (1 *g*_s_ + µg, **E**–**G**). Thanks to the electron-dense structure, PA precipitates are visible as spherical black dots and were mainly observed in the cytosol (**A** and **E**). Some precipitates were detected near statoliths, inside the vacuoles (**D**) and the nucleus (**F**). No precipitates were found either in the cell wall (**B**) or in the statoliths (**C**). Arrows indicate examples of PA precipitates in various cellular components. C: cytosol; CW: cell wall; N: nucleus; S: statoliths; V: vacuole. Bar scales indicate 2 µm.

To monitor the changes in the content of cytoplasmic free calcium, we used a method specific for calcium precipitation in plants namely the pyroantimonate (PA) precipitation method^23,24^. PA precipitates have the advantage of being electron-dense structures with a round shape, facilitating their observation and counting on ultrathin sections using transmission electron microscopy (Fig. 2) and their detection from image analysis. The presence of calcium ions in PA precipitates was confirmed by X-ray microanalysis (Supplementary Fig. S2).

The observation of electron microscopy images revealed that PA precipitates were mainly distributed in the cytosol of root statocytes (Fig. 2A,E). Some precipitates were also observed inside cell organelles as nuclei (Fig. 2A,F) and vacuoles (Fig. 2D). No precipitates were observed in statoliths (Fig. 2C,G) or in the cell wall (Fig. 2B). This intracellular distribution was observed in all conditions including the simulated gravity onset and removal conditions. The low number of precipitates in each organelle did not allow for the statistical evaluation of the changes in PA precipitate repartition.

The counting of PA precipitates using image analysis (Table 2) showed that their density was similar in statocytes of roots grown for 40 h in microgravity conditions (µ*g*) and under simulated gravity 1 *g*_*s*_ (P = 0.812). On the other hand, a significant four-fold increase was observed in the 10 minutes simulated gravity condition (µ*g* + 1 *g*_s_, P = 0.024) compared to the µ*g* condition. A significant two-fold increase was also observed in the 10 minute microgravity condition (1 *g*_*s*_ + µ*g*, P = 0.019) compared to the 1*g*_s_ condition. These increases clearly show that both gravistimulation onset and removal triggered an increase in the number of PA precipitates, suggesting an increase in free cytosolic calcium in root statocytes.

**Table 2:** Density of calcium precipitates in root statocytes, expressed as numbers of precipitates by a 500 µm^2^ protoplasm unit area.

Condition	Density of precipitates (number per 500 µm² area)	Number of analysed cells
1 *g*_*s*_	9.2^a^ ± 1.3	14
1 *g*_*s*_ + µ*g*	16.9^b^ ± 2.7	14
µ*g*	9.8^a^ ± 2.3	12
µ*g* + 1*g*_*s*_	44.1^b^ ± 11.9	8

*B. napus* seedlings were grown for 40 h in simulated gravity (1 *g*_s_); 39 h 50 min in simulated gravity followed by 10 minutes in microgravity (1 *g*_s_ + µ*g*); 40 h in microgravity (µg); 39 h 50 min in microgravity followed by 10 minutes in simulated gravity (µ*g* + 1 *g*_s_). Data are means ± se. By column, means followed by a same letter are not significantly different at a 5% level using a Student-Newmans Keuls test.

### Statolith positioning in root statocytes

**Figure 3:**
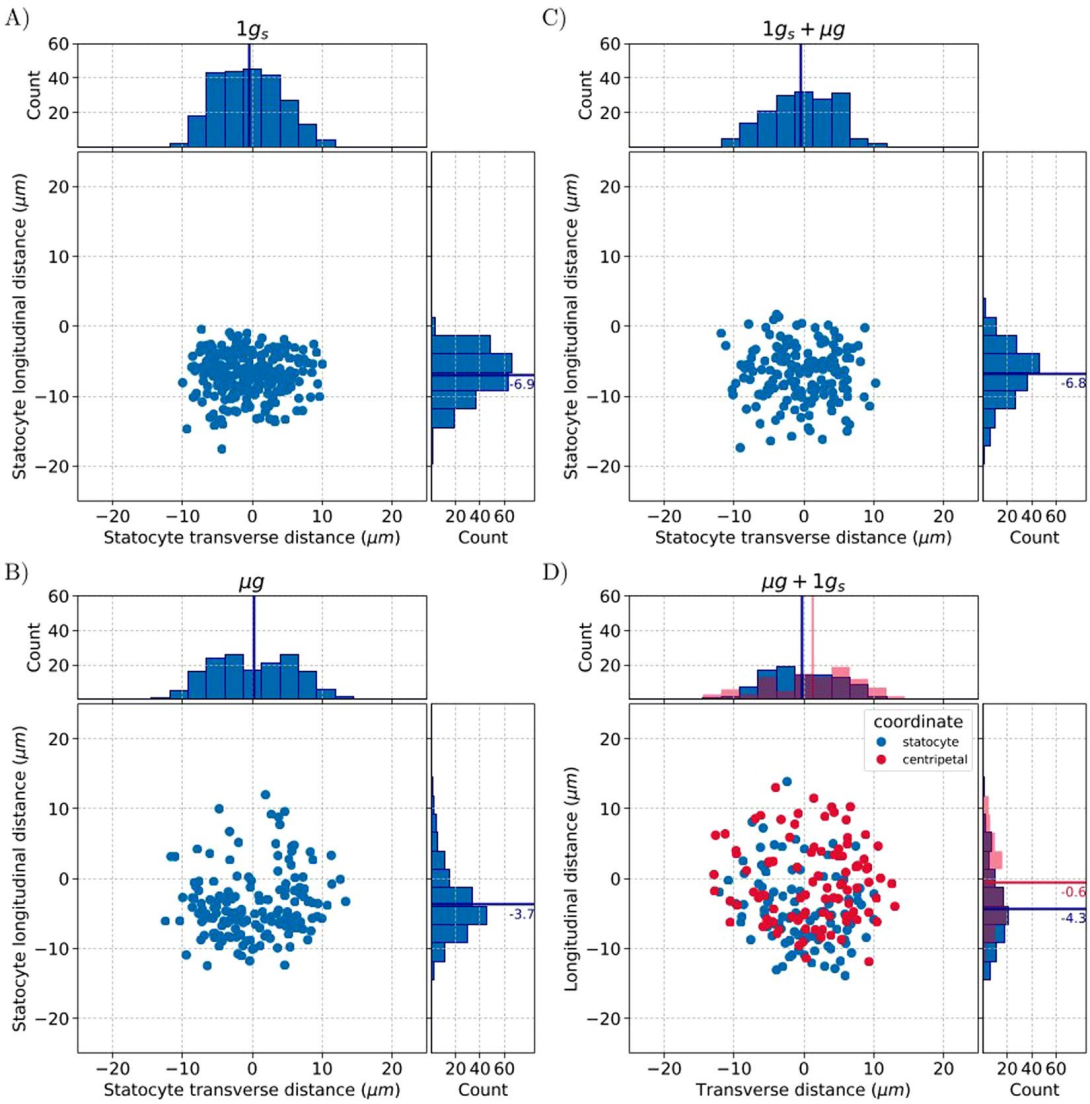
Statolith positioning within root statocytes. The position of each statolith is indicated in blue and is given relative to the statocyte centroid (0,0) along the cell transverse and longitudinal directions. Data are shown for each condition: 1 *g*_s_ (**A**), µ*g* (**B**), 1 *g*_s_ + µ*g* (C), µ*g* + 1 *g*_s_ (**D**) and are accompanied by a histogram of counts along each direction. In the µ*g* + 1 *g*_s_ (**D**) condition, red spots indicate the position of statoliths along the centrifugation force (centripetal coordinates). Solid lines and values indicate the means of counts for each condition.

Our space experiment allowed us to acquire and analyse the coordinates of a total of 664 statoliths (Supplementary Fig. S3). No change in root orientation was reported after the 10 minutes of gravistimulation onset and removal (Fig. 1D). We therefore considered the final root orientation (and thus of statocytes) to be representative of the root orientation before the 10 minutes of simulated gravity onset and removal. We then transformed each statolith position from the image coordinate system to the statocyte coordinate one in which the origin is located at the centroid of the cell, and the y-axis is aligned with the longitudinal direction of the cell (*i.e*. the longitudinal axis of the root). For each experimental condition, the position of all statoliths in the statocyte coordinate system was represented (Fig. 3A–D; Supplementary Fig. S3).

As expected in *Brassica napus* roots growing in simulated gravity (1 *g*_s_), a polarized structure of statocytes with a nucleus in the shootward side of the cell was observed. A voluminous vacuole occupied the cell (Fig. 2). All statoliths were localized in the rootward half of each statocyte (no statolith was positioned above zero along the cell longitudinal direction; Fig. 3A). Statoliths were equally distributed along the transverse section (55% in left side, 45% in right side), resulting in all roots being in the same direction than the simulated gravity vector (Fig. 1D). Surprisingly, most of the statoliths of roots growing in microgravity (µ*g*) were also located in the rootward part of each statocyte, with only 18% of statoliths above the position of the cell centroid (Fig. 3B). However, the longitudinal distribution of statoliths in microgravity showed a long distribution tail for higher values, while in simulated gravity the distribution along the longitudinal cell direction was visually close to a Gaussian distribution (count histograms in Fig. 3A,B). In addition, the mean relative position of statoliths along the longitudinal direction from roots grown in microgravity (c.i. 95%: −4.4 ≤ µ ≤ −2.9) was significantly different from the mean position of statoliths from roots grown in simulated gravity (c.i. 95%: −7.2 ≤ µ ≤ −6.4; p-value < 0.001). Thus, the mean statolith position in *Brassica napus* roots growing in microgravity was from 2.0 to 4.3 µm closer to the statocyte centroid compared to roots growing in simulated gravity. Moreover, the standard deviation was a useful indicator to show that in the population of statoliths in simulated gravity, there was less of a spread than in the population of statoliths in microgravity (s.d. = 3.1 µm in 1 *g*_*s*_ and 4.6 µm in µ*g*).

Statolith positioning in roots that were subjected to 10 minutes of simulated gravity onset and removal (µ*g* + 1 *g*_s_ or 1 *g*_s_ + µ*g*) were compared to their respective initial conditions (µ*g* or 1 *g*_s_) (Fig. 3). No statistical difference was found in the mean positioning of statoliths: neither in the longitudinal, nor in the transverse direction in cells of roots submitted to 10 minutes of microgravity (1 *g*_s_ + µ*g*), compared to their controls (1 *g*_s_). Similarly, statolith positioning after 10 minutes of simulated gravity (µ*g* + 1 *g*_s_) were not statistically different from that observed in microgravity (µ*g*, Fig. 3C). Given the higher variances and the size of samples, the minimum displacement that would have been detected is 1.0 µm after 10 minutes of microgravity and 1.8 µm after 10 minutes of simulated gravity in this experimental design. These results suggest that both simulated gravity onset and removal induced a statolith displacement of low amplitude. Statoliths coordinates were also transformed along the centrifugation force (centripetal coordinates) but still no significant displacement toward the centrifugation force direction was observed (Fig. 3D). The dissymmetry along the transverse direction of statolith distribution in µ*g* + 1 *g*_s_ results from the higher number of roots oriented rightward in this condition (39% on the left side, 61% on the right side; Fig. 3D count histogram). In microgravity, statoliths were equally distributed along the transverse section (51% on the left side, 49% on the right side).

In conclusion, the statolith positioning along the root longitudinal axis was statistically different between microgravity and simulated gravity statocytes. No difference in statolith positioning was found after the 10 minutes of simulated gravity onset and removal, suggesting that any expected displacement was of low amplitude or that not all statoliths were displaced.

## Discussion

Root statocytes are polarized cells where the endoplasmic reticulum is located near the cell wall, mainly towards the root apex, and the nucleus is maintained at the shootward end by the cytoskeleton^3^. Inclination and centrifuge experiments undoubtedly showed that statoliths settle in the direction of the gravity vector independently of the root statocyte polarity. In microgravity, though, the positioning of statoliths reaches a stable state close to the nucleus in lentil roots^17,25,26^. Statolith grouping near the cell center in microgravity was also confirmed from 3D reconstruction of root statocytes of white clover^27^. Consistent results were found in the current study where statoliths were more spread out in microgravity and their mean position was significantly closer to the cell center. However, it can be noticed that statolith positioning in microgravity was surprisingly close to statolith positioning under simulated 1 *g*_s_ with only 18% of statoliths towards the nucleus. One hypothesis is that the intracellular space available for statolith displacement was limited by the surrounding organelles and, notably, by the presence of vacuoles that occupied most of the intracellular space. The presence of a large central vacuole in stem statocytes and its close interaction to statolith dynamics is known as stem statocyte^28^. In root statocytes, vacuoles are expected to be smaller and less present than in surrounding cells, including meristematic cell, cells of the lateral columella and cells of the outer root cap^2,29^. Large vacuoles in root statocytes may be either a characteristic of rapeseed or a consequence of cultivation in microgravity. This observation has already been noted in microgravity with root statocytes from soybean seedlings^30^. Another hypothesis is that the motor of statolith displacement towards the nucleus in microgravity is weaker in rapeseed as already reported in lentil seedlings^17^.

Our analysis also highlights that neither the onset of simulated gravity after microgravity (µ*g* + 1 *g*_s_) nor the removal of simulated gravity for 1 *g*_s_ roots (1 *g*_s_ + µ*g*) induced a detectable or significant statolith displacement compared to the initial position of statolith. In fact, no displacement or rather low displacement was observed. Statolith velocity reported after changes of inclination is in the range of 0.3–1.2 µm min^−1^ for *Lepidium*^31^. In maize, it decreases from 19.1 µm min^−1^ to 3.7 µm min^−1^, a few seconds to 30 seconds after change of inclination^32^. Statolith sedimentation has been reported to be over after 400 seconds in *Arabidopsis* roots^33^. Accordingly, the mean difference of 3.2 µm observed between statolith positioning in microgravity and simulated gravity should disappear when microgravity roots are submitted to ten minutes of simulated gravity. However, one should keep in mind the variability of root apex orientation in microgravity relative to the centrifuge rotor, which prevents the use of the microgravity condition as a consistent starting point. When root apex orientation relative to the centrifuge rotor is homogeneous (1 *g*_s_ and 1 *g*_s_ + µ*g* gravity condition), it is easier to highlight a displacement of statoliths. Statolith velocity from stimulated gravity to microgravity was previously estimated at close to 0.15 µm min^−1^ by Perbal and Driss-Ecole in lentil seedlings^17^. According to this range of velocity, statolith displacement after 10 minutes of microgravity is sufficient to remove contacts with another cell component, but still too low for being detected in this study. All these observations sustain that in rapeseed a very low displacement of statoliths occurred or that only a small number of statoliths were displaced. The latter is in accordance with the concept of “lead statoliths” where statoliths at the forefront are more prone to be displaced during the first minutes following a change of inclination^34^. According to previous experiments done in microgravity, the combination of threshold values for gravity sensing with statolith position in microgravity sustains that low displacement of statoliths of similar amplitude (below 1 µm) is sufficient to trigger the downstream transduction pathway of gravity sensing^35^.

The pyroantimonate (PA) method was successfully used to study cellular and subcellular localization of calcium in gravistimulated organs. Previous comparisons between microgravity and 1 *g*_s_ grown roots highlighted a decrease of the density of PA precipitates in microgravity, especially in the nucleus and cytosol while the density of PA precipitates increases in the vacuole^25,36^. Similar observations were made in response to simulated microgravity using clinostat^26^. Experiments including short gravistimuli in oat coleoptiles grown on Earth showed PA precipitate relocalization within 30 minutes in the bending region after a change of inclination^36^. Here, the density of PA precipitates was similar in both the simulated gravity and the microgravity conditions, representing conditions with stable levels of gravity during the whole experiment. Following the gravistimulus induced by the centrifuge, we observed a significant four-fold increase of the PA precipitate density, whereas a very slight statolith displacement was observed. These observations hypothesize that the establishment of contacts between the statoliths and any close cell component may be of importance for triggering the release of calcium. PA precipitate density also increased by a two-fold factor after removal of the simulated gravity *i.e*. after removal of contacts and nullifying the weight of statoliths. This observation indicates that the gravity transduction pathway might be linked to statolith motion and reposition while discarding mechanisms based on the detection of a force applied by statoliths, confirming previous works^9^.

In conclusion, this study suggests that statolith displacements of very low amplitude were sufficient to trigger the release of intracellular free calcium after simulated gravity onset. This release of intracellular free calcium is expected to be the cause or/and the consequence of the triggering of the gravity transduction pathway. Interestingly, this result was also observed after simulated gravity removal, indicating that the weight of statoliths may not be essential for gravity sensing. This observation provides valuable information about the variable that could be sensed by the statocytes. A first hypothesis could be the sensing of statolith repositioning through contact establishment or removal with another cell component. A second hypothesis is the sensing of statolith motion using an intracellular network like the actin cytoskeleton. *In vivo* experiments coupling statolith tracking with an indicator of the gravity transduction pathway such as calcium and pH monitoring should help in disentangling the gravity sensor functioning. Identifying the reservoir of intracellular calcium that is mobilized in the gravity signal transduction is also a primary objective for upcoming studies.

## Methods

### Kubik facility and PolCa hardware

The space experiment, called *PolCa*, was performed on the Kubik facility, located in the International Space Station (ISS). Kubik is designed for automatic microgravity experiments and was built by the COMAT company (Toulouse, France) for the European Space Agency. The Kubik facility consists of a small controlled-temperature incubator with a centrifuge, which enables simultaneous 1 *g* control samples to run with microgravity samples (Fig. 1A). When the centrifuge was activated, a simulated gravity acceleration (*g*_*s*_) was generated perpendicular to the main axis of the culture chamber (Fig. 1B,C) with an intensity equal to the Earth gravity (g) (1 *g*_*s*_ = 1 *g*) at the position of the seeds. Specific experimental units (EUs,) can be inserted and removed on the centrifuge or in the incubator.

**Figure 4:**
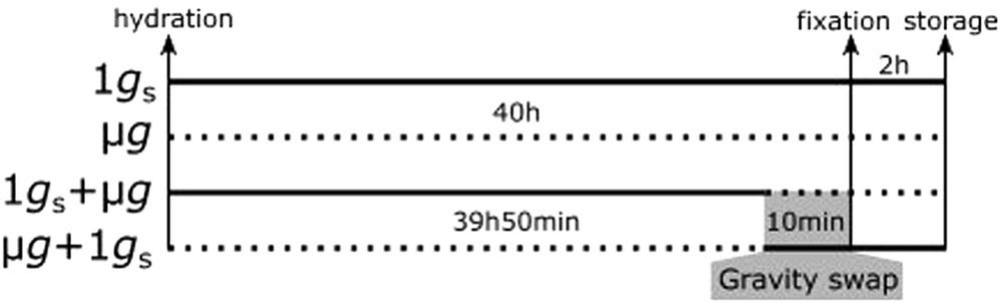
*PolCa* experimental timeline as performed in the Kubik facility. The cosmonaut positioned four experimental units (EUs) on a centrifuge and four EUs in microgravity conditions, triggering simultaneously an automatic water injection in the CCs. The water injection, leading to seed hydration, was considered as the starting point of the experiment. 39 h50 after hydration in simulated gravity, two EUs were transferred to microgravity conditions by the cosmonaut and correspond to 1 *g*_s_ + µ*g* samples. At the same time, the two EUs initially in microgravity were placed on the centrifuge, corresponding to µg + 1 *g*_s_ samples. The time period of gravistimulation onset and removal corresponds to 10 minutes for each CC. Two EUs were left on the centrifuge, corresponding to control simulated gravity (1 *g*_s_) and two others were kept in microgravity conditions, corresponding to control microgravity (µ*g*). In each EU, fixative solutions were automatically injected from the paunch connected to the CC, leading to the fixation of all seedlings in their growth conditions at 40 h after hydration. EUs were left on the centrifuge or in microgravity for 2 h and then stored at 6 °C until being opened on Earth. The solid lines indicate the time period in simulated gravity. The dotted lines indicate the time period in microgravity.

Eight specific experimental units (EUs) designed and built by the COMAT Company, according to our specifications, were used for the *PolCa* experiment. Each EU includes a culture chamber (CC) dedicated to seedling hydration, connected to a pouch of water for the germination activation and to one or two fixative pouches (Fig. 1B). The water and fixative solutions were automatically injected in the CCs at the appropriate time according to the predetermined timeline (Fig. 4). Each CC contains a plastic seed support able to receive 7 *Brassica napus* seeds and it is then sealed with a transparent plastic cover.

### Pre-flight ground preparation

After cleaning the CC, an appropriate piece of Whatman paper was placed in the plastic seed support of the CC. A double-sided adhesive tape was added onto the upper side of the paper in order to maintain seeds and avoid any physical displacement due to centrifuge acceleration and deceleration. Seven dry seeds of *Brassica napus* var Pau (Biogemma, Toulouse, France) were layered out and oriented in such a way that the embryonic root was perpendicular to the longitudinal axis of the CC (Fig. 1B). Seeds were then covered by a piece of cellulose sponge in order to facilitate germination after hydration. The prepared supports with seeds were stored in the darkness at 22 °C during 8 days.

The 2 fixatives used for the experiment were prepared in Toulouse (France). The fixative dedicated to the analysis of statolith positioning contained 3% paraformaldehyde (v/v) (Sigma, ref. P6148) and 0.5% glutaraldehyde (EMS, ref. 16210) in 0.1 M sodium phosphate buffer (Acros Organics, pH 7.2). The fixative dedicated to the precipitation of free calcium contained 3% potassium pyroantimonate (v/v) (EMS, ref. 20220), 0.5% glutaraldehyde and 2% paraformaldehyde in 0.1 M sodium phosphate buffer (pH 7.2). The fixatives were stored for 8 days at 4 °C during their transport. The fixative pouches were then filled at Baikonur (Russia) with each fixative and, one day before the launch, the support with seeds was inserted in the CC. All materials were then stored at 22 °C.

### Experimental flight timeline

The *PolCa* experiment was launched on March 26, 2009 on a Soyuz rocket on flight TMA-14, during the Expedition 19 mission and was back on earth with Soyuz capsule on the April 7, 2009. The experiment was performed on the ISS in the Kubik instrument from April 3 to April 6, 2009.

Upon their insertion in the Kubik facility, the 8 CCs were stored an ambient temperature until the start of experiment (Fig. 4). The experiment started when cosmonaut Yuri Lonchakov inserted the EUs in the Kubik facility, triggering automatic water hydration in all EUs at the same time. Four EUs were placed on the Kubik-centrifuge dedicated for simulated gravity control (1 *g*_s_) and 4 EUs were placed within the same Kubik incubator, in order to be exposed to microgravity conditions (µ*g*). After 39 h50 of growth, the cosmonaut stopped the centrifuge and swapped the position of 4 EUs: two EUs initially placed on the centrifuge were placed in microgravity (1*g*_s_ + µ*g* condition). Simultaneously, 2 EUs initially in microgravity were placed on the centrifuge (µ*g* + 1 *g*_s_ condition). Forty hours after hydration, including or not the 10 minutes of gravistimulation onset and removal, fixatives were automatically injected without the interruption of experimentally controlled gravity condition. All experimental steps were performed around 26 °C (Supplementary Fig. S5). After 2 hours of chemical fixation, the 8 EUs were retrieved from the Kubik facility and stored at 6 °C until their opening at the laboratory (France).

### Root apex embedding and sectioning

Upon reception, EUs were carefully opened and inspected to check fixation status. Pictures of all CCs were taken and seedling roots were collected. The first 5 mm root apices were sampled and individually immersed in culture multiwell plates containing fresh fixative for 30 minutes. Root apices were then rinsed with 0.1 M sodium phosphate buffer (pH 7.2) under stirring (3 × 15 min) and progressively dehydrated using successive immersions in graded series of ethanol (30 min each on ice) at 25% [vol/vol], 50%, 60%, 80%, 95%, 100%. Each root apex was infiltrated progressively in LR White resin (Sigma-Aldrich, 94188-59-7) through successive incubations in graded series of LR White at 30%[vol/vol], 50% and 75% and finally in pure LR White for 12 hours per step. Samples were then embedded in 100% LR White using flat embedding capsules (VWR, 100488-336) ensuring proper apex orientation and finally hardened at 60 °C for 48 h.

For the analysis of statolith positioning, semi-thin longitudinal sections of the root cap (0.5 µm of thickness) were realized using a microtome. The sections were then stained with toluidine blue 0.1% (in sodium carbonate 2.5%, pH 10.5), and were observed under an optical microscope (Zeiss, Germany). For the analysis of calcium precipitation, ultrathin longitudinal sections of the root cap (around 100 nm of thickness) were realized using an ultramicrotome, dried overnight, post-stained with uranyl acetate for 20 minutes and rinsed with distilled water. All ultrathin sections were observed under a transmission electron microscope coupled to an X-ray microanalysis system (Philips CM12).

### Image analysis

After the opening of the EUs, pictures of all CCs were taken and analysed using ImageJ software^37^ (v.1.5, https://imagej.nih.gov/ij) to determine the root length and the orientation of the root apex.

The root cap of *Brassica napus* seedlings contains seven cell layers from the quiescent center to the root apex. Statolith positioning was analysed on the two central cells of layer 4 and layer 5 corresponding to a total of 4 cells; Supplementary Fig. S6). The location of these four cells in the central columella make them good candidates for being statocytes^4,38–40^. Images at high magnification (100×) were used to determine statolith positioning within the statocytes. Images were first contrasted and converted to binary images. Cell membrane and statoliths were then manually segmented to extract the coordinates of the centroids of these cellular components. As the root orientation (and by extension statocytes orientation) was not conserved during the embedding steps, images at intermediate magnification (40×) were used to extract the section orientation. Coordinates along the section orientation were rotated relative to the root longitudinal direction or relative to the centrifuge centripetal axis using the pictures of the CCs previously described.

The PA method was proved useful to evaluate the impact on the cytoplasmic free calcium content and localization in response to gravity alterations or inclination^41–43^. The PA method is highly sensitive, allowing the detection of the precipitation of cytoplasmic free calcium at concentration down to 10^−6^ M, which is above cellular concentration in the cytosol (10^−7^ M range) and under cellular concentration in the nucleoplasm and vacuole^44^ (10^−3^ M range). During signal transduction, concentration can vary locally in the cytosol above 10^−6^ M and, thus, induces formation of PA precipitates^44^. The PA method also maintains a direct relationship between [Ca^2+^]_c_ and [Ca^2+^] recovered from precipitates, linear for concentration in the mM range^41,42^. Thus, the number of PA precipitates is a good proxy of the quantity of [Ca^2+^]_c_ and can be used for comparisons between our different conditions.

The analysis of pyroantimonate (PA) precipitates was done on statocytes of the layer 4 of the root cap. The quantity of PA precipitates was determined in each cell from the transmission electron microscope images taken at a magnification of 3810×. To compare cells with different sizes, the density of pyroantimonate precipitates was determined for an area of 500 µm² corresponding to the average statocyte surface.

### Statistical analysis

For all traits considered, as root length, density of precipitates and statoliths coordinates, the data were analyzed by using a one-way analysis of variance (ANOVA) with the PROC GLM procedure of SAS (v9.3, SAS Institute Inc, Cary, NC, USA, 2006). The homogeneity of variances was tested using the Levene’s test. When the assumption of homogeneity of variance was rejected or when unbalanced data were performed between groups, a Welch’s Anova was used instead of the usual Anova to test for differences between group means. Means obtained for the different gravity conditions were separated using a Student-Newman-Keuls multiple comparisons test when a main effect was determined to be significant at a 5% level.

Estimation of the least significant displacement of statoliths that could have been detected was done by generating *i* theoretical samples having same size and standard deviation as experimental samples. Draws were done on a theoretical Gaussian distribution and an increasing difference in the mean of each condition was generated by step of 0.1 µm (*i* = 10 000 per step). Fischer t-test was used to test the difference between samplings on each draw. Least significant displacement was reported when Fischer t-test detected a difference in at least 80% of the draws (Statistical power 1 − β = 0.8 and α = 0.05).

## Electronic supplementary material

Supplementary Material
