# Supplementary material for: Both gravistimulation onset and removal trigger an increase of cytoplasmic free calcium in statocytes of roots grown in microgravity
Source: Sci Rep. 2018 Jul 30;8:11442. doi: 10.1038/s41598-018-29788-7 (PMC6065396; doi:10.1038/s41598-018-29788-7)
Supplement: Supplementary file 1 — Supplementary Material [file 41598_2018_29788_MOESM1_ESM.pdf]

Both gravistimulation onset and removal trigger an increase of cytoplasmic free calcium in statocytes of roots grown in microgravity

François Bizet, Veronica Pereda-Loth, Hugo Chauvet, Joëlle Gérard, Brigitte Eche, Christine Girousse, Monique Courtade, Gérald Perbal & Valérie Legué

Additional information

Supplementary Fig. S1: Root apex distribution at the end of the experiment. An angle of 0° indicates the centripetal direction (*i.e.* direction of simulated gravity g_s when applied). sd: standard deviation.

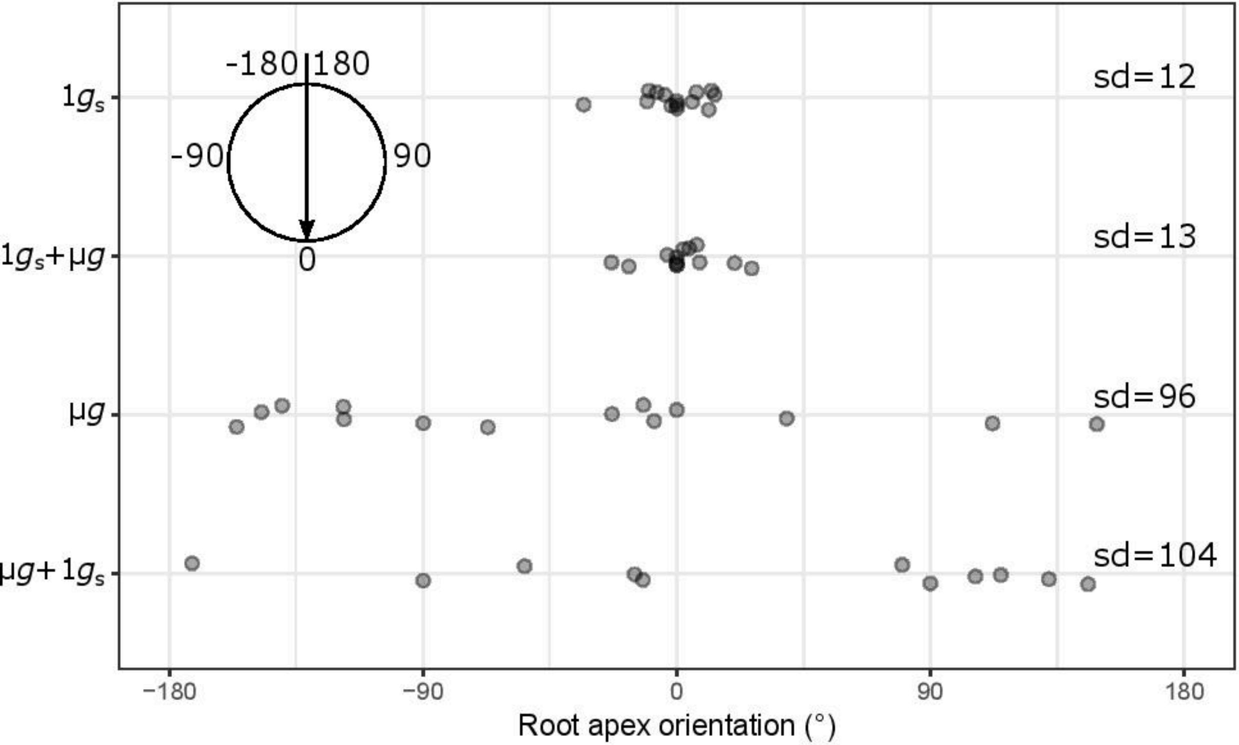

-180
180
-90
90
0
sd=12
sd=13
sd=96
sd=104
$1g_s$
$1g_s+\mu g$
μg
$\mu g+1g_s$
−180
−90
0
90
180
Root apex orientation (°)

Fig. S2: Representative X-ray spectrum after targeting of the PA precipitates in root statocytes. The applied method gives a quality spectrum identifying the characteristic peaks of calcium (Ca) and antimonate (Sb) elements found in the PA precipitates. A characteristic peak is observed at 3620 eV (dashed line) resulting from the combination of the Sb-Lα (3600 eV) and the Ca-Kα (3690 eV) emissions.

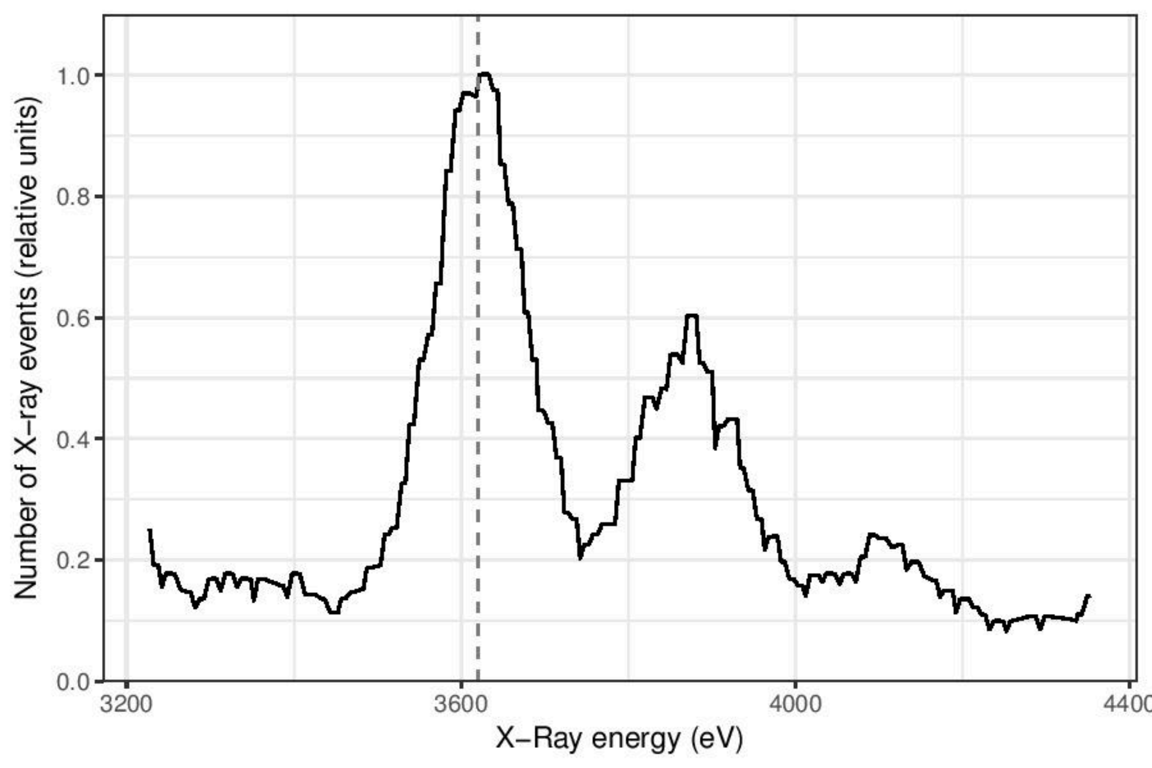

1.0
0.8
0.6
0.4
0.2
0.0
Number of X−ray events (relative units)
3200
3600
4000
4400
X−Ray energy (eV)

Fig. S3: Statoliths positioning within root statocytes. The position of each statolith is given relative to the statocyte centroid along the cell transverse and longitudinal direction. Low values along the longitudinal direction indicate positions closer to the root apex. Data are shown for each condition: $1g_s$ (A), µg (B), $1g_s$+µg (C), µg+$1g_s$ (D). This figure is a reproduction of Fig.3 with colors added in each condition to indicate individual roots.

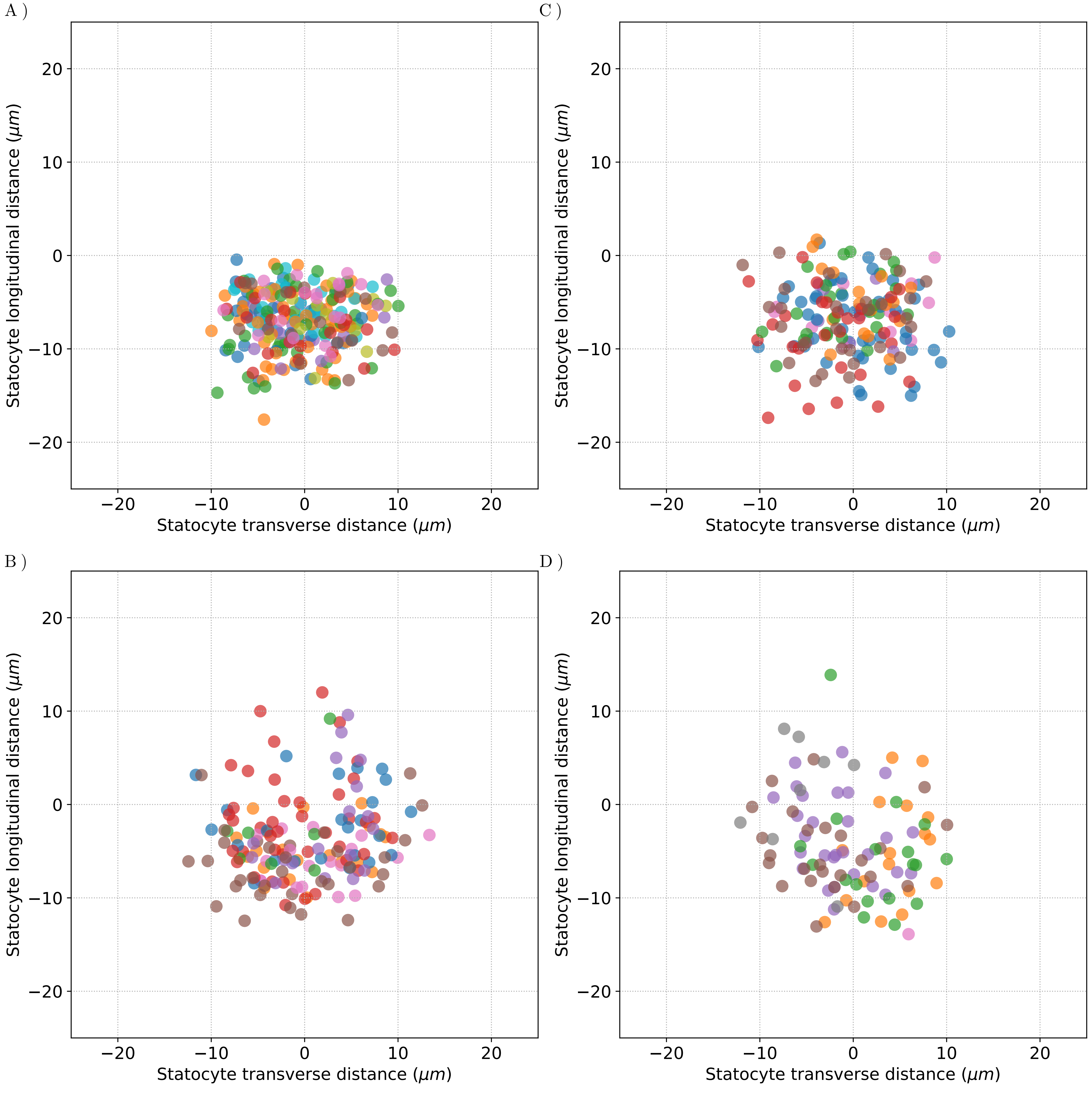
A)
B)
C)
D)
Statocyte longitudinal distance (μm)
Statocyte transverse distance (μm)
20
10
0
−10
−20

Fig. S4: Problems caused by variable root orientation relative to the gravity vector when quantifying statolith displacement. Theoretical examples for *Brassica napus* root statocytes (with low statoliths even in microgravity, see Fig. 3B). (A) When statolith positions are shown along the statocyte polarity, the variability of orientation of the gravity vector hide the displacement of statoliths toward the statocyte cell wall. The mean position along the statocyte polarity in microgravity (black) is thus not different from the mean position after gravistimuli when several individuals are taken (mean of colors). (B) When measurements are presented along the simulated gravity vector, statocytes having variable orientations before gravistimuli (colors) present various statoliths mean position along the vector. When polarity is equally spread in all directions the mean statoliths position before gravistimulus is expected to be 0, and the gravistimulus should lead to negative values (not shown here for readability purposes). Thus misleading higher values are observed compared to the previous statocyte polarity coordinate system.

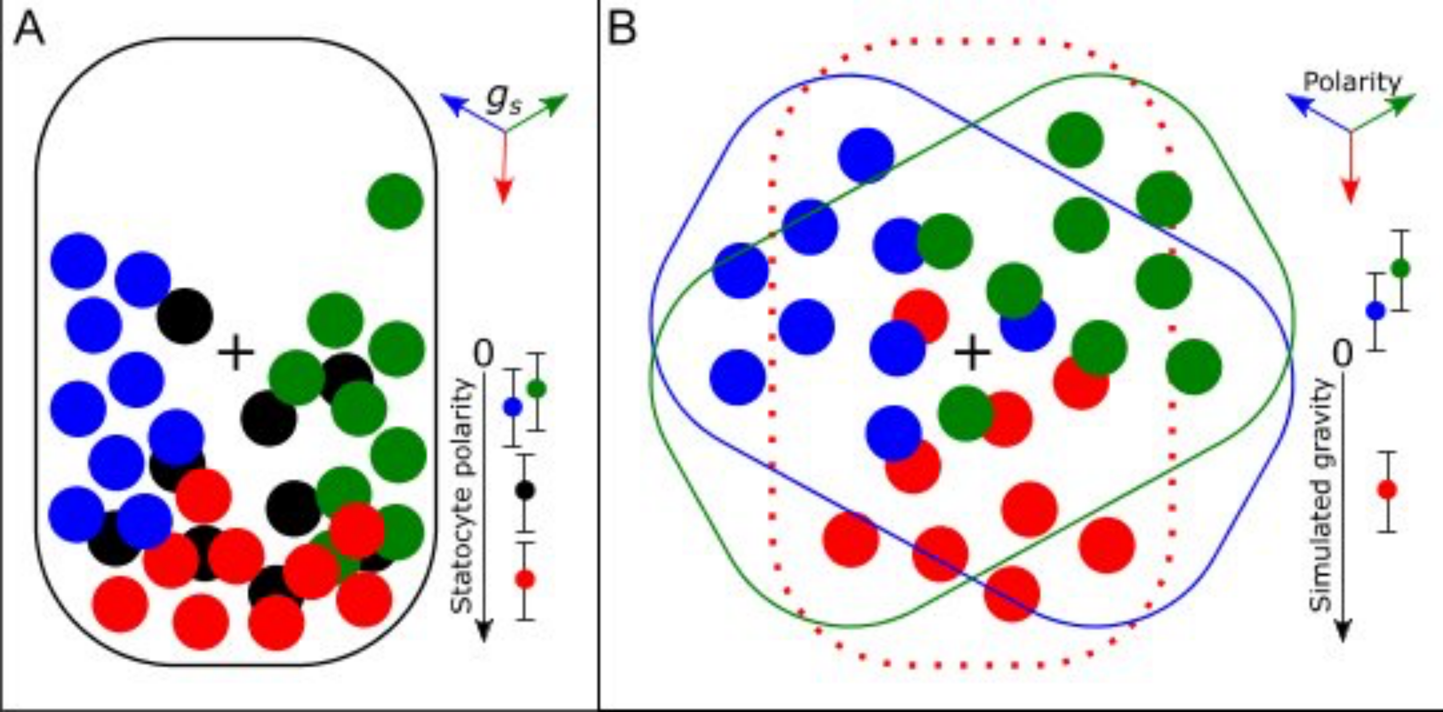

A
g_s
0
Statocyte polarity
B
Polarity
0
Simulated gravity

Fig. S5: Temperature profile measured inside the experimental units (EUs) during the *PolCa* experiment. The curve starts at the time of seed hydration. From left to right, dashed lines indicate the period of gravistimulation onset and removal condition (bold line), the end of sample fixation (2h from gravistimulation onset and removal) and the landing of the Soyuz rocket. EUs were then transferred at 6°C before shipping to laboratory for analysis.

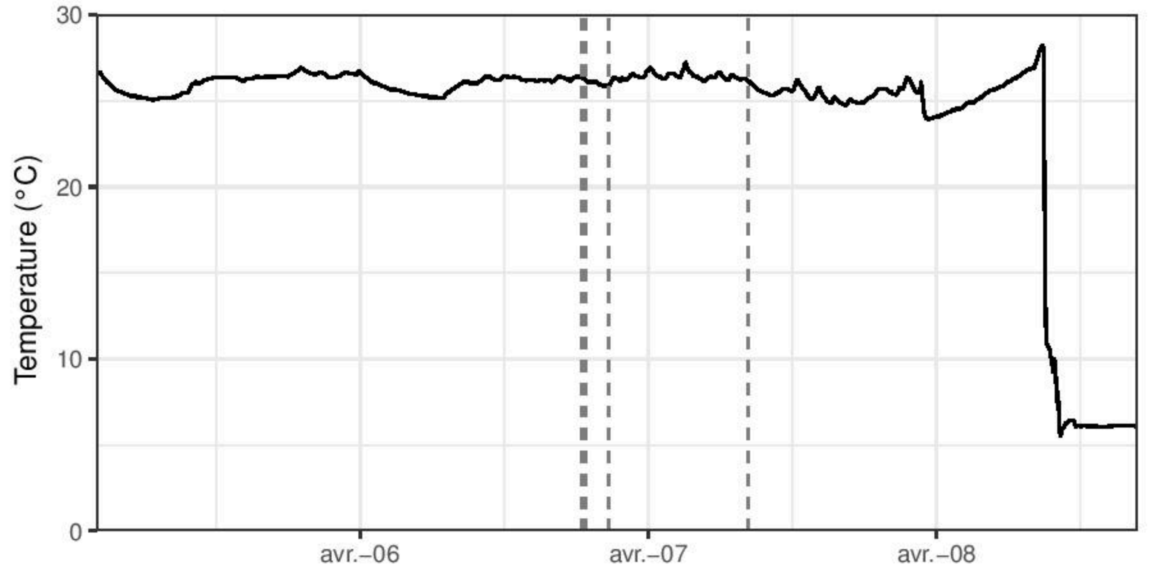

Temperature (°C)
30
20
10
0
avr.–06
avr.–07
avr.–08

Fig. S6: Representative root cap structure of *Brassica napus* seedling at 40h after hydration. Median longitudinal semi-thin root section stained with toluidine blue indicates a root cap with 7 cell layers, numbered L1 to L7 from the Quiescent Centre (QC). The QC is in white. The black line indicates cells located in L4 and L5 layers of the central columella used for PA precipitates analyses (L4 only) and statolith positioning analyses (L4 and L5). Scale bar =20 µm.

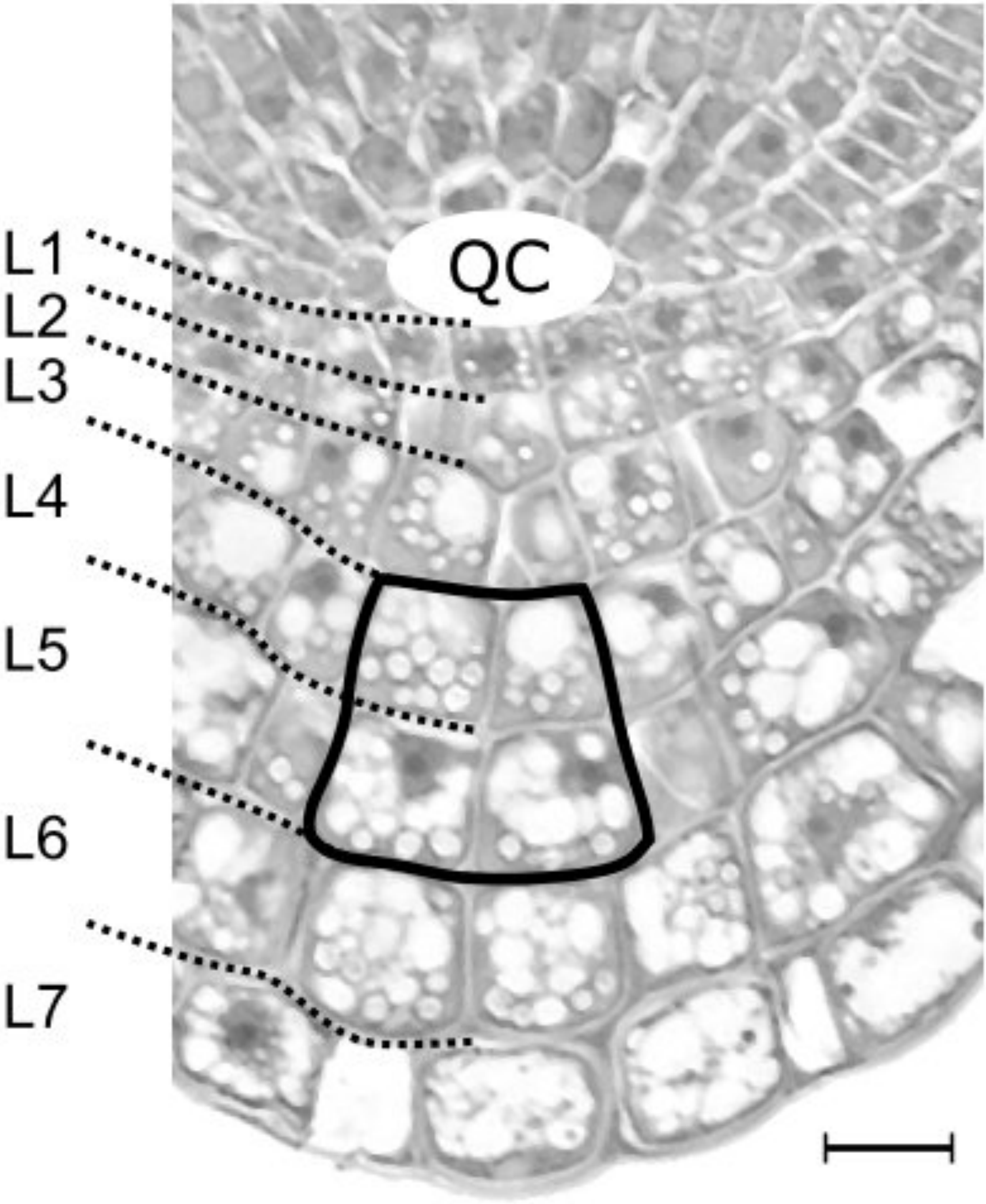

L1
L2
L3
L4
L5
L6
L7
QC
